# Computational Intelligence-Based Disease Severity Identification: A Review of Multidisciplinary Domains

**DOI:** 10.3390/diagnostics13071212

**Published:** 2023-03-23

**Authors:** Suman Bhakar, Deepak Sinwar, Nitesh Pradhan, Vijaypal Singh Dhaka, Ivan Cherrez-Ojeda, Amna Parveen, Muhammad Umair Hassan

**Affiliations:** 1 Department of Computer and Communication Engineering, Manipal University Jaipur, Dehmi Kalan, Jaipur 303007, Rajasthan, India; sumanbhakar2016@gmail.com (S.B.); deepak.sinwar@gmail.com (D.S.); prof.dhaka@gmail.com (V.S.D.); 2 Department of Computer Science and Engineering, Manipal University Jaipur, Dehmi Kalan, Jaipur 303007, Rajasthan, India; nitesh.pradhan943@gmail.com; 3 Allergy and Pulmonology, Espíritu Santo University, Samborondón 0901-952, Ecuador; ivancherrez@gmail.com; 4 College of Pharmacy, Gachon University, Medical Campus, No. 191, Hambakmoero, Yeonsu-gu, Incheon 21936, Republic of Korea; 5 Department of ICT and Natural Sciences, Norwegian University of Science and Technology (NTNU), 6009 Ålesund, Norway

**Keywords:** disease severity, deep learning, machine learning, Parkinson’s disease, diabetic retinopathy, Alzheimer’s disease, CNN, J0101

## Abstract

Disease severity identification using computational intelligence-based approaches is gaining popularity nowadays. Artificial intelligence and deep-learning-assisted approaches are proving to be significant in the rapid and accurate diagnosis of several diseases. In addition to disease identification, these approaches have the potential to identify the severity of a disease. The problem of disease severity identification can be considered multi-class classification, where the class labels are the severity levels of the disease. Plenty of computational intelligence-based solutions have been presented by researchers for severity identification. This paper presents a comprehensive review of recent approaches for identifying disease severity levels using computational intelligence-based approaches. We followed the PRISMA guidelines and compiled several works related to the severity identification of multidisciplinary diseases of the last decade from well-known publishers, such as MDPI, Springer, IEEE, Elsevier, etc. This article is devoted toward the severity identification of two main diseases, viz. Parkinson’s Disease and Diabetic Retinopathy. However, severity identification of a few other diseases, such as COVID-19, autonomic nervous system dysfunction, tuberculosis, sepsis, sleep apnea, psychosis, traumatic brain injury, breast cancer, knee osteoarthritis, and Alzheimer’s disease, was also briefly covered. Each work has been carefully examined against its methodology, dataset used, and the type of disease on several performance metrics, accuracy, specificity, etc. In addition to this, we also presented a few public repositories that can be utilized to conduct research on disease severity identification. We hope that this review not only acts as a compendium but also provides insights to the researchers working on disease severity identification using computational intelligence-based approaches.

## 1. Introduction

Early and accurate diagnosis of diseases is essential for the right treatment. In addition to accurate and rapid diagnosis, the severity identification using computational intelligence-based approaches is becoming popular and challenging nowadays. Traditional computational approaches (i.e., classification) are mainly focused on solving two-class classification problems, i.e., positivity or negativity of disease, or presence or absence of certain values. However, nowadays, with the advancements in deep learning technologies, one can easily diagnose the disease and its severity. Most of the work on severity identification is based on recent deep-learning-based models. The training of these models depends on the labeling of disease severity levels by expert personnel. However, the process of multi-class manual labeling is quite tedious, time-consuming, and non-quantitative [1].

**Figure 1:**
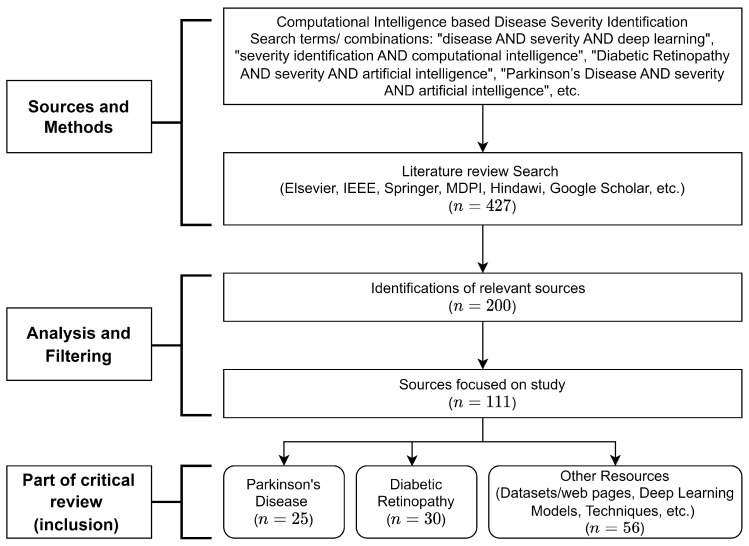
Strategy for inclusion of sources for this study.

In this paper, the problem of severity identification is addressed with the help of multi-class classification. A comprehensive review of various research articles concentrating on disease severity identification using computational intelligence-based approaches is presented. Research articles focused on the severity identification of Parkinson’s Disease (PD) and Diabetic Retinopathy (DR) are mainly considered for this study. We followed the PRISMA statement to prepare this review on the severity identification of diseases using computational intelligence-based approaches. The search terms/combinations to search sources for this study followed search phrases such as “(disease AND severity AND deep learning)”, “(severity identification AND computational intelligence)”, “(Diabetic Retinopathy AND severity AND artificial intelligence)”, “(Parkinson’s Disease AND severity AND artificial intelligence)”, etc. The search strategy followed by the identification and analysis of sources for this study is also depicted in Figure 1. In addition to this, we briefly surveyed a few articles on the severity identification of some other diseases, i.e., COVID-19, Knee Osteoarthritis (KOA) [2], Autonomic Nervous System Dysfunction (ANSD) [3], Tuberculosis [4], and Sepsis [5], etc. It is evident that radiology is widely used for the diagnosis of various critical diseases. Some computational approaches also consider radiological images for disease identification. Radiology is one discipline of medicine that uses imaging technologies to diagnose diseases [6]. Radiology is divided into two main classes, viz. Diagnostic Radiology and Interventional Radiology [7]. Diagnostic radiology provides structures inside the body, whereas interventional radiology is associated with minimally invasive procedures.

**Figure 2:**
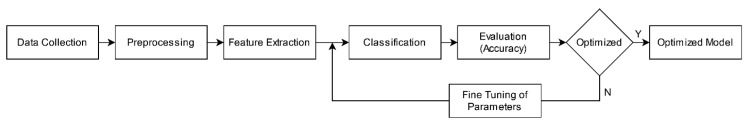
Process of optimizing a classification model for disease identification.

Due to the recent advances in deep learning and machine learning, the potential of computational approaches regarding the recognition of complex patterns from radiological images has increased to a great extent. Nowadays, the integration of computational approaches and radiological imaging technologies is gaining tremendous popularity and becoming an active research area. Undoubtedly, future clinical decision support systems and monitoring systems will be equipped with state-of-the-art artificial intelligence. It is observed that plenty of deep-learning- and machine-learning-based research work has been carried out on radiological imaging. Deep-learning-based disease identification follows several steps, viz. data collection, labeling, classification, and model evaluation. These models can be optimized by fine-tuning the parameters, as depicted in Figure 2.

In the case of machine-learning-based disease identification, the non-imaging data values and efficient algorithms play an important role in decision support systems. Machine-learning and deep-learning techniques have numerous applications in the medical domain. The work embodied in this paper mainly focuses on diagnosing Parkinson’s disease, Diabetic Retinopathy (DR), and some other diseases (infectious diseases, tuberculosis, COVID-19, sepsis, etc.) using computational approaches. The subsequent sections will highlight some of the work conducted by researchers to diagnose these diseases. In short, the major contributions of this paper are highlighted as follows:

- In-depth analysis of several recent pieces of work for disease severity identification using computational intelligence-based approaches.
- A comprehensive discussion on the challenges and issues of each approach for severity identification.
- Classification of several works according to major disease types such as Parkinson’s Disease and Diabetic Retinopathy.
- Presentation of several public repositories for conducting disease severity identification research.

The remainder of this paper is organized as follows. Section 2 and Section 3 discuss some of the work related to detecting the severity of Parkinson’s Disease and Diabetic Retinopathy, respectively. Works based on severity identification of a few other diseases, i.e., COVID-19, autonomic nervous system dysfunction, tuberculosis, sepsis, sleep apnea, psychosis, traumatic brain injury, breast cancer, knee osteoarthritis, and Alzheimer’s disease, are briefly presented in Section 4. A few public repositories are depicted in Section 5. Finally, Section 6 presents concluding remarks along with future directions for severity identification using computational intelligence-based approaches.

## 2. Severity Identification of Parkinson’s Disease

Movement disorders caused by PD may not remain the same in different patients. Thus, it is essential to develop an automated tool to evaluate a patient’s gait. Xia et al. [8] presented a novel gait evaluation approach (known as “dual-modal attention-enhanced deep learning network”), which not only distinguishes between normal gaits and PD gaits but also computes the severity of PD by quantification of gaits. The system is capable of modeling both left and right gaits separately. Multiple 1D vertical ground reaction force (VGRF) signals achieve the segmentation of left and right samples. A CNN-LSTM-based dual-modal attention-enhanced network was utilized to analyze the gait movements on the gait dataset [9] with two severity levels, viz. Hoehn and Yahr (H&Y) and the Unified Parkinson’s Disease Rating Scale (UPDRS). Their architecture utilizes an input with the dimensions B × 150 × 9 × 1, where B indicates the batch size of samples, 150 indicates the period of a sample, and 9 indicates the number of VGRF signals. Their CNN consists of three layers in which every convolution operation is followed by the ReLU activation function for feature extraction. However, pooling is not incorporated due to the limited data samples. After the last convolution, the output of the feature map comprises dimensions of B × 150 × 9 × C3. Using flattening, the feature map 9 × C3 is converted into a tensor, i.e., C4, which was fed to an attention-enhanced LSTM (AE-LSTM). The AE-LSTM concatenates the branches and passes them to the fully connected (FC) layer. Finally, the severity of PD is achieved using probability distribution by mapping the output of FC using a SoftMax classifier. Experimental results claim 99.01% accuracy in classifying PD patients into different severity levels.

Pereira et al. [10] have reviewed several papers to predict PD at the earliest stage. After reviewing the papers, the authors have concluded that there are still many problems that need to be addressed, so they proposed image processing techniques to address these existing problems. For this experiment, handed datasets are utilized, collected from Brazil University. It contains the meander and spiral images gathered through the handwritten exam and 92 handwritten exams conducted on healthy people (control group) and PD patients. Handwritten Trace (HT) and Exam Template (ET) features are extracted through the blurring method. The feature extraction technique is applied to compare and evaluate both the HT and ET features. The Support Vector Machine (SVM) with some modifications, Naïve Bayes (NV) technique, and Optimum path forest (OPF) pattern recognition methods are used for the severity classification. The experimental results show 67% accuracy in identifying the precise class to predict the stage of the severity. As per the amount of information concerned for PD identification, meander images represent more information than spiral images. Although they presented an automated system that diagnosed the PR at an early stage, the performance can be improved by considering large as well as consistent datasets.

Prashanth et al. [11] addressed the fact that if PD disease is detected at an early stage, it can be cured by the proper therapies and medicines. In this regard, they utilized Single-Photon Emission Computed Tomography (SPECT) along with 123I-Ioflupane to diagnose the PD disease at an earlier stage on the PPMI database. The dataset contains the Striatum Binding Ratio (SBR) value of 179 normal people and 369 PD patients in the initial stage. The logical regression is applied for the calculation of the significant numerical features. The visualization of each SBR feature is calculated through histograms. The notched plots mark the patients separately in normal, PD, and early-stage categorization. The classifications and prediction have been acquired through the Support Vector Machine (SVM) and Logistic Regression (LR). The SVM uses a linear kernel to classify the decision boundary through by input features. The binomial logistic regression model uses the logit transformation method to develop the prediction model to predict the risk factor in PD patients. The experimental results report that the SVM classification method has achieved 96.14% accuracy and 95.03% specificity for the classification of PD patients. Although this system provided high performance and distinguished early PD patients from normal patients, the system can be enhanced through the Scans Without Evidence of Dopaminergic Deficit (SWEDD) and other validation approaches.

**Figure 3:**
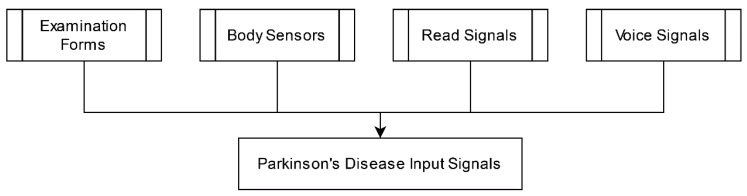
Various inputs to Parkinson’s Disease diagnosis.

Parkinson’s Disease can be identified on various input signals, as depicted in Figure 3. In this regard, Cernak et al. [12] proposed a model to identify voice characteristics to predict the PD patient’s information. They utilized the read Voice Quality (VQ) datasets by Kane (2012) and Laver (1980). They covered the five non-model vocalizations, viz. creaky, breathiness, falsetto, harsh, and tense. To study the vocalization features, the Spanish database contains the speech recording detail of PD patients and a healthy control group. With the help of statistical measures, the authors differentiated the model and non-model vocalization. They computed the probability of the vocalization features through a machine-learning-based approach. The Euclidean distance calculates the similarity of the model in PD, and the alignment of the non-model is calculated through the inverse distance. The vocalization analysis section is computed through the Deep Neural Network (DNN). Further, the binary classification method was utilized to identify the probability of a specific vocalization class. They also applied the acoustic model for the phonic configurations. The experimental results reported the characteristics of PD patients: the composition of a maximum of 30% of breathy voice and a minimum of 12% of harsh voice. The system provided the accuracy of the vocalization speech based on the voice quality, but analysis of the speech was limited due to available datasets.

Lahmiri et al. [13] also proposed a method to detect PD through voice patterns. They utilized the 195 vowels and voices data set comprising 147 PD-affected and 48 healthy patients. The Wilcoxon and ROC techniques were used to identify eight different patterns. The well-established SVM classification technique was applied to classify the PD patient and the healthy one. The system reported a 92.21% accuracy, 82.79% specificity, and 99.63% sensitivity. Although this automated system provided a good performance through voice patterns only, the researchers may combine some other parameters for the identification of PD patients at an early stage because voice is not the only symptom that characterizes PD.

Ertuǧrul et al. [14] presented a machine-learning model to detect PD disease at an earlier stage. Initially, the data are collected from the gaitpdb datasets that contain information about healthy people and PD patients. Eight sensors are placed under the foot for 2 min, and the recorded sensor information is converted into the LBP domain and processed through shifted 1D-LBP. The LBP signal value lies between 0 and 255, matched with a special and distinct pattern formed through the shifted 1D-LBP signal. Then, the histogram technique illustrated the 256 different signal patterns according to their corresponding signal. The statistical features such as correlation, entropy, and skewness are computed through the 1D-LBP histogram sensor. The classification and design features were processed through the machine-learning approach. The experiment evaluation on 10-fold cross-validation reported an accuracy of 88.89% and a sensitivity of 0.89. The authors implemented the proposed system on biomedical information, and in addition to this, some other symptoms such as speech may be considered in the future.

Marek et al. [15] stated that PD detection at the earliest age is crucial because there is no accurate method to detect PD. Either motor symptoms or non-motor symptoms can be detected through PD diseases. They proposed an automated multi-modal feature and machine-learning techniques based on non-motor symptoms for detecting PD. Based on biomarkers, the feature description is processed through the REM sleep Behavior Disorder Screening Questionnaire (RBDSQ) and CerebroSpinal Fluid (CSF). The Wilcoxon sum test is applied for the feature analysis. The PD classification is achieved through SVM, random forest, and logistic regression. The experimental result reported a 96.0% accuracy for the tested dataset.

Acharya et al. [16] differentiated PD patients from normal persons by drawing movements. They investigated handwriting markers for muscular movements and interpretation of other activities of the patients. To experiment with this model, the dataset was categorized into two parts, i.e., 20 healthy and 57 PD patients. The data pre-processing was achieved through five different score vectors. The Normalized Velocity Variability (NVV) is applied to identify the speed of the pen of the subject. They applied the NVVALL score to focus on healthy and PD patients. The receiver operating characteristic (ROC) was observed to be 0.9354. The UPDRS score represented the writing behavior of PD patients on the Hoehn (H) and Yahr (Y) scale. Naïve Bayes, Adaboost, and logistic regression methods were applied for the PD classification. The experimental results reported the highest accuracy of 90.90% through Naïve bays and the lowest accuracy of 86.36% through the SVM classifier.

Nilashi et al. [17] presented a new automated method to predict and monitor PD disease patients with characteristic motor and total UPDRS. Clustering was applied to form a cluster with similar characteristics and merge similar features into one cluster. Thus, in the output, different clusters were created of different sizes. A self-organizing map (SOM)-based cluster approach effectively handled the large datasets and provided similar clusters. The R^2^ method was utilized to evaluate the value of the SOM. In addition, the PCA method was applied for the feature analysis of the cluster approach. Further, the deep belief network was also applied to identify PD patients better. The RMSE method was applied to find the exact and accurate information about PD patients. They also included the SVR [18] and ANFIS [19] learning techniques and presented an accuracy of 89.4%.

Sztaho et al. [20] proposed a method to detect the severity level of Parkinson’s disease through speech signals. To implement this method, the authors used the Hungarian speech database that consists of the speech signals of 51 patients. The severity of patients was classified according to the Hoehn (H) and Yahr (Y) scales. The sound card was utilized to record the speech of patients. The feature extraction technique was utilized to categorize speech, such as pause ratio and speech speed. The authors implemented this method using two types of detection methods, viz. binary classification and regression. The classification method was processed by the K-Nearest Neighbor (K-NN) method and SVM. They utilized two types of regression methods, viz. linear regression and support vector regression. The Root Mean Square Error (RMSE) was used to evaluate the performance of the regression method. The binary classification method reported an overall accuracy of 83.56% for the read text, 85.11% for the speech signal, and 84.62% for both.

Xia et al. [8] proposed a dual model based on the deep-learning method to detect the characteristics of Parkinson’s disease from the gait signals. The left and right gaits were recorded by the VGRF tool. The severity level is identified with the help of the Hoehn (H) and Yahr (Y) scales. They applied an N-size vector for feature extraction and selection through this vector gait cycle detection, which is processed by fixing the N = 150. The dual-mode consists of two-channel levels for processing separate signals. The VGF gait signals are first passed through the two-layer CNN model to understand the features of gait signals, followed by LSTM for temporal features. Further, they utilized the attention method, which provided meaningful information on the subject that can be accessed with the help of a score. A Fully Connected layer (FC) was incorporated to combine both left and right gait signals, followed by final classification through the SoftMax layer. The efficacy of the model was measured using a five-fold cross-validation approach. The model experimentally reported an accuracy of 99.31% and a sensitivity of 99.23%.

Park et al. [21] compared the performance of the PD diagnosis system through SVM with the two methods, viz. Multiple Layer Perceptron (MLP) and Radial Basis Function Network (RBN). Seventy-four-year-old data are utilized to implement this method, and the signal Electromyograph (EMG) is recorded through the AgCI conductor. In the pre-processing stage, signals are firstly filtered into 3 to 10 Hz by a type-2 filter followed by Fast Fourier Transformation (FFT) to identify the same frequency band of the tremor. After these steps, EMG signals are classified into two stages, viz. experienced and visual signal to detect the exact tremor status. The MLP network consists of the input layer, hidden layer, and output layer, and it is used to reduce the overfitting issue in the datasets. The status of tremors is detected through −1 and 1. On the other hand, the radial basis function utilized the fuzzy c-mean clustering method to identify the initial stages of the cluster. Overall, 81.14% accuracy was reported using the SVM classification of tremor status.

Hariharan et al. [22] presented an intelligent system based on a hybrid model. They initially incorporated the Gaussian mixture method as a pre-processing step to remove the unwanted noise present in the dataset. They also utilized two types of feature reduction methods, viz. PCA (Principal Component Analysis) to identify the hidden features presented in the datasets and LDA for mapping 22 features into a one-dimensional space. General Regression Neural Network (GRNN), Probabilistic Neural Network (PNN), and SVM were utilized for the severity classification of PD. The promising classification was reported based on the cross-validation method.

On the other hand, Balaji E. et al. [23] proposed a machine-learning model that can assist clinicians in detecting the stages of PD through gait information. Gait information provides all mobility information about healthy people and PD-affected people. This model is trained and tested with the public datasets based on the gait pattern provided by Physionet. VGRF is placed under the foot to provide gait information through different sensors. The feature extraction process is achieved using statistical and kinematic feature extraction approaches. The statistical feature extraction process is used to identify the four levels of PD through H and Y scales. It created a 16 × 166 matrix based on the sensor and subject-level PD severity. In contrast, the kinematic features were used to identify PD patients’ steps, swing time, and speed. A 10-fold cross-validation is adopted in which 90% of data are used for training purposes and the remaining for testing purposes. Decision Tree (DT), SVM, Bayes, and Ensemble classifier were utilized for the classification. Experimental evaluation reported that the Decision Tree (DT) classifier has the highest accuracy of 99.04%, the sensitivity of 99.06%, and the specificity of 99.08%.

Kim et al. [24] presented a novel approach based on CNN to detect the severity rate of Parkinson’s disease by performing tremor quantification from raw datasets. For experimental evaluation, 92 PD patients’ tremor sensor datasets were collected using a wrist sensor device as wearable equipment. A neurologist was provided with the information on PD on four-level severity, i.e., normal to severe, based on the unified Parkinson’s disease rating scale (UPDRS). In addition, they designed a neural network to assess the severity in PD patients. In this network, 2D images are used as input for the convolution layer, and a 3 × 50 convolution filter combines both local and sensor information. They processed the input signals computed by the wrist sensor in the form of gyroscope signals and accelerometer signals. Experimental evaluation depicted a classification accuracy of 85%.

Oung et al. [25] addressed that the existing system does not differentiate between people infected with Parkinson’s Disease (PD) and healthy people. Therefore, to handle this issue, they proposed a multi-class classification system to classify PD severity levels (low, mid, high) and a healthy control group. For experimental evaluation, datasets of 65 persons of different ages were collected from the Neurology hospitals and the severity level in Hoehn (H) and Yahr (Y) was rated through the UPDRS measure. The dataset signal is assorted through two stages, i.e., motion and speech-based signals. The speech signals were recorded through the Motion Node Bus (MNB) from the IMU wearable device, and the speech signals were recorded through the audio sensor, i.e., a headset placed at 5 cm away from the mouth. The authors acquired the Empirical Wavelet Transform (EWT) to decompose the motion signals to find the approximate information from the detailed information, and the Empirical Wavelet Packet Transform (EWPT) was developed to decompose the speech signals. The EWPT method uses Fast Fourier transform (FFT) to obtain the exact frequency, i.e., lies between 0 and π. Feature extraction was processed through the Hilbert transform based on amplitude and frequency. Extracted features are categorized into three groups: speech signals, motion signals, and a mix of motion and speech. They employed Probabilistic Neural Network (PNN), Extreme Learning Machine (ELM), and K-Nearest Neighbor (kNN) for the classification. Experimental evaluation reported an accuracy of 90% on classification using an Extreme Learning Machine (ELM) for both motion and audio signals.

Recent studies analyzed that it is hard to diagnose PD at an earlier stage. Many remote detecting tests were utilized to detect the PD severity and realized that variables in gait signals could easily distinguish PD patients from healthy ones. In this regard, Cantürk et al. [26] proposed a system to detect PD patients’ severity using gait signals. Their system was trained and tested with 306 publicly available signals with 93 PD patients and 73 healthy subjects based on different categories. The gait system was measured through Ultraflex Computer Dyno Graphy (UCDG) with eight sensors placed under the foot. The Fuzzy Recurrence Plots (FRP) convert the signals into texture representations for both PD and healthy patients. Further, AlexNet was applied to extract the deep features, followed by implementing SVM and k-Nearest Neighbor (kNN) for binary and multi-class classification. The experimental result of the kNN method reported an accuracy of 99%, whereas the SVM reported 98%.

Zhao et al. [27] presented a machine-learning method to detect the severity level of PD from the gait data. This is the hybrid technique consisting of both Long Short-Term Memory (LSTM) and a Convolutional Neural Network (CNN) to recognize the spatial time-based pattern through the gait data. The hybrid model has five convolution layers and two layers of LSTM to detect the severity rate in PD patients. The authors acquired two convolution layers of 5 × 5, in which the first layer is mapped with 32 features and the second one is mapped with 64 features. LSTM and CNN are trained and tested on the PhysioNet [28] dataset. The pre-processing and L2 normalization were applied to reshape the datasets into 100 × 19 × N (N =“Ga:13592, Si:7744, Ju:11734”). Further core parameters of LSTM were transformed to achieve better classification results into four levels, viz. normal (severity 0), severity 2, severity 2.5, and severity 3. Final classification was achieved using the SoftMax layer. The model reported 98.70% accuracy for the first dataset, 98.41% for the second dataset, and 98.88% for the third dataset. However, this method provided better accuracies in PD detection, and this model is the baseline for detecting the PR disease.

An automated machine-learning-based method is proposed to detect and identify the level of severity of Parkinson’s disease from the gait data by Maachi et al. [29]. They employed a Deep Neural Network with the help of a 1D convolution Neural Network. This algorithm has divided the information into two parts, viz. Parkinson’s and a control group. For the experiment, publicly available datasets are used and cited from the PhysioNet. The datasets contain 93 patients with Parkinson’s disease and 73 patients in the control groups. The Vertical Ground Reaction Force (VGRF) based on 18-1D signals provides the information of a recorded walk with the foot sensors positioned below the foot. The VGRF signal is divided into datasets into m-parts that are based on subject categorization. Further, these parts are the input of the proposed method of DNN. The DNN method is processed with two parts, viz. 18 parallel 1D and a fully connected network. The feature extraction is processed through the 18 1D-CNN. The Parallel 1D network has taken input from the VGRF signal and processed it through the four convolution layers, which are fully connected. Further, this layer has extracted the features used to help categorize the PD and control groups. The output layer generates one neuron to detect the disease and five neurons to classify the level of severity that were categorized into five classes based on some criteria. This method reports an accuracy of 98.7% in detecting the severity and 85.8% accuracy in the classification of the severity level.

Prashanth et al. [30] addressed different stages of PD as a very important factor in a medical decision. The subject’s disordering features were measured by UPDRS, but it does not give information about the PD stage. In this paper, they proposed a new model based on machine-learning to detect the PD and different stages of PD (early, normal, and moderate). This hybrid model supports SVM, AdaBoost, and RUSBoost-based and ordinal logistic regression (OLR) classifiers. It utilized the Parkinson’s Progression Markers Initiative (PPMI) datasets with 197 healthy and 434 PD subjects. The statistical analyzer is used to classify the features into three categories based on a filter. They used classification algorithms such as random forests, SVM, and logistic regression to classify the PD stages. The validation of the performance was measured by the 10-fold cross method. The experimental results indicated that AdaBoost reports the highest detection accuracy of 97.46% for the normal PD subject, and SVM reports 98.04% for the early stage of PD detection. Although automated detection improves the stage of PD, there is a need to address more stages for PD patients.

Prashanth et al. [31] also presented a prediction model based on machine-learning to distinguish healthy and early PD patients. The dataset utilized for the experiment is from the Parkinson’s Progression Markers Initiative (PPMI). They further applied the Patient Questionnaire (PQ) to analyze the dataset. In PPMI, data are arranged in the longitudinal format, so they performed the record and subject-wise cross-validations. The dataset is divided into 90% training sets, and the remaining are test sets. To remove the redundancy and select the appropriate features, they have used three different selection methods, viz. Wilcoxon rank, Least Absolute Shrinkage and Selection Operator (LASSO), and Principal Component Analysis (PCA). The Wilcoxon rank method is acquired for the significant features through the sum test. The LASSO method is also applied to shrink the datasets, and the PCA method is the reduction approach used for decomposing the multivariate datasets into one manner format. The authors have processed the logistic regression, SVM, random forests, and boosted trees for the classifications. The experimental results indicated 96.50% accuracy using SVM through the subject-wise validation.

Aydın et al. [32] presented the Hilbert–Huang Transform (HHT) method to detect the severity of Parkinson’s Disease (PD) from the gait pattern. The datasets are utilized from the PhysioNET [28], and the signals, such as step swing time, are measured through the VGRF sensor. The authors applied three types of feature selection techniques, i.e., the filter approach, the wrapper approach, and the embedded approach. The filter approach is used to identify the common characteristic of the training datasets. The wrapper feature selection approach is applied for mapping with relevance and extracting the optimal features, and the last approach is applied to check the performance of the features. They also applied the feature creation method, and a 10-fold cross-validation approach checks the performance of this method. The regression tree classification approach is processed to distinguish PD patients from healthy ones. The experimental results showed that the accuracy of the proposed system is 98.79%, sensitivity is 98.92%, and specificity is 98.61%. The performance analysis of some PD identification approaches is depicted in Table 1. On the other hand, a systematic review of AI-based approaches for the diagnosis of PD is presented by Saravanan et al. [33].

### Discussion

As stated earlier, the movement disorders caused by Parkinson’s Disease are not uniform in all patients. Deep-learning models play crucial roles in developing automated tools for evaluating a patient’s gait. It is obvious that to cure any disease, its detection must take place at the early stages. To detect PD at an early stage, both artificial intelligence and machine-learning-based techniques are contributing to a great extent, e.g., feature extractions and pattern recognition from motor symptoms, voice pattern recognitions, etc. Plenty of work has been carried out to identify PD at an early stage, but this field is still in its infancy stage. It is observed that very few works are available on PD identification using non-motor symptoms, and the availability of PD datasets is not adequate to develop automated models. The researchers may consider these issues while developing an automated model for the detection of PD at an early stage with excellent efficiency.

## 3. Severity Identification of Diabetic Retinopathy

Excessive glucose growth in the blood causes diabetes that subsequently harms other components of the human body, i.e., eyesight loss, kidney malfunctioning, nerve failure, damage to blood vessels, etc. This excessive amount of glucose leads to damage to the retina’s blood vessel, which is the main cause of Diabetic Retinopathy (DR) disease. Blurriness, color difficulty, floaters, and dark vision are early symptoms of DR disease. It has become one of the major reasons globally for visual losses. Timely diagnosis and subsequent treatment of its several stages/severities can save visual loss to some extent. Several computational models are presented by plenty of researchers for the detection of DR from fundus images. Shankar et al. [35] presented a novel automated model called HPTI-v4 (Hyperparameter Tuning Inception-v4) DR detection from color fundus images. Initially, the contrast of fundus images is enhanced using Contrast Limited Adaptive Histogram Equalization (CLAHE) [36] followed by histogram-based segmentation. HPTI-v4 then processes the segmented images for feature extraction followed by a Multi-Layer Perceptron (MLP) classifier. Experimental results on the MESSIDOR (Methods to Evaluate Segmentation and Indexing Techniques in the field of Retinal Ophthalmology) DR dataset exhibited that HPTI-v4 outperforms other state-of-the-art deep-learning models (i.e., ResNet [37], GoogleNet [38], VGGNet-16 [39], VGGNetCOVID-19 [39], VGGNet-s, AlexNet [40], Modified AlexNet, and DNN-MSO). The dataset consists of 1200 posterior pole eye fundus images that were mainly classified into four classes, viz. normal, stage-1 (images with some microaneurysms), stage-2 (image with both microaneurysms and hemorrhages), and stage-3 (images with high microaneurysms and hemorrhages). In addition to HPTI-v4, 10-fold cross-validation was used to subdivide the dataset into training and testing sets; and Bayesian optimization was employed for selecting an optimal set of hyperparameters. The proposed HPTI-v4 obtained the highest accuracy of 99.49% as compared to other models under consideration.

Wang et al. [41] presented a hierarchical multi-task framework based on deep learning for simultaneously detecting DR features and severity levels. Severity levels in DR are characterized by the presence of various signs in the fundus images. DR severity identification becomes easier if the DR disease-related signs are present in the fundus images. Earlier, Wang et al. [42] investigated the feasibility of diagnosing DR severity levels and the presence of DR-related features. Their hierarchical multi-task framework consists of two main tasks, viz. severity diagnosis of DR and identification of DR-related features. Their architecture consists of one backbone squeeze-and-excitation (SE) network [43] for feature extraction and two neural networks (one for DR-related feature extraction and the other for severity detection). To validate their framework, an experimental evaluation was conducted on two independent test sets, followed by a grader study to compare the performance of the proposed framework with experienced ophthalmologists. Results depicted that the proposed model was able to improve the performance of traditional machine-learning-based approaches.

Torre et al. [44] developed a new method to detect diabetic retinopathy using a deep-learning classifier. For the system’s good performance, they categorized the retinal images according to the level of severity. To experiment with this model, the EyePacs dataset [45] from Kaggle was utilized. The ophthalmologists classified the images into different criteria based on the grading scale. The authors also applied the deep-learning model modifications to classify the retinal images. They used the optimal retina images whose diameter size is 640 pixels. The Rectified Linear Unit (ReLU) is applied for the activation function with an epoch size of 30. The multi-class classification model is used to obtain better identification of the disease as well as severity levels. The experimental results reported a specificity of 91.1% and a sensitivity of 90.8%. The main advantage of this model is classifying the five-severity levels of DR disease and identifying the score levels of each class.

Shankar et al. [46] proposed a Synergic Deep-Learning Model (SDL) to classify the severity level of diabetic retinopathy fundus images. The model utilized the MESSIDOR [47] dataset, which contains 1200 color fundus images. The first step was to apply the pre-processing in which each image was converted into RGB format. Then the segmentation was performed through a histogram to fetch the green color of the image for further information. The SDL model has processed the classification to classify the DR image into different stages. Different performance matrices, such as accuracy, sensitivity, and specificity, are used to evaluate the system. The model experimentally proved 99.28% accuracy for the classification, 98.54% sensitivity, and 99.38% specificity.

Nowadays, few smartphone-based systems help in performing the retinal screening of diabetic patients. Still, the accuracy of DR identification is based on the quality of the image and the region of the view. Therefore, the smartphone system must consider a highly compact design to provide accurate information. In this regard, Hacisoftaoglu et al. [48] presented a new system to detect DR based on a smartphone-based system through a deep learning approach. The system uses transfer learning approaches such as GoogLeNet [38] and ResNet [37]. The validation of the experiment has been processed through different datasets, i.e., MESSIDOR and EyePacs. The experimental result reported an AUC of 0.99, a sensitivity of 98.2%, a classification accuracy of 98.6%, and a specificity of 99.1%. On the other hand, Son et al. [49] developed a new model that helps to identify the abnormality in DR patients based on retinal images. They utilized three datasets for the validation of the approach; 103262 images from 309786 were used to develop the model, and for the testing, other external datasets were used. Finally, the MESSIDOR dataset [47] was used for comparison purposes. The deep-learning model has been applied to classify the abnormality in retinal images. The classification output has oscillated from 0 to 1, which shows the probability of finding the existence of abnormalities. The experimental evaluation reported an ROC value of 96.2% to 99.2%. The proposed deep-learning model not only categorizes the finding by accuracy but also calculates the salient features of the images.

A new automated method based on deep CNN for detection of DR is proposed in [50]. They utilized two datasets to validate their study: the EyePACS and MESSIDOR-1 & 2. The pre-processing has been performed in both online and offline stages. In the online stage, the image is cropped in the desired shape, followed by the removal of the black border of the image, whereas in offline mode, the pre-processing has been processed by the augmentation method. The results of the model show better accuracy on the same public datasets compared to other existing algorithms. Moreover, the suitable preliminary process for screening larger numbers of patients for an automated system is batch processing and minimum assumption time. The efficient screening process helps to obtain the model’s best results. The model was found to enhance the 0.92 AUC for the MESSIDOR-2 dataset [51] with a sensitivity of 81.02% and a specificity of 86.09%.

The automated NAS (Neural Architecture Search) machine-learning model [52] predicts the DR patients with no and severe stages of DR disease. To train and validate the model, a Kaggle dataset comprising the information of 3662 images was used. Out of 3662 images, 3113 images were used for the training data sets, and the remaining were used for the testing datasets. Harikrishnan et al. [52] first applied the pre-processing steps to remove the unwanted noise and other information. They resized the image in a particular format, then applied the Gaussian filter to improve the image quality. The NAS acquired the RNN (Recurrent Neural Network) to add more functions with different combinations to obtain the optimal solution. The accuracy of the model was reported to be 75%. To develop this model, the learning rate was set as 0.0001, and the initial weight was chosen as the net image weight. The authors observed that the model obtained the minimum accuracy when including the dense layer without a pre-processing stage. The proposed model was validated through the existing database based on E-Ophtha Exude. This model also reported a sensitivity of 76.6% and a specificity of 77.1%.

Washburn et al. [53] proposed a new system design to detect the retinal image at the earliest phase. The model utilized the public retinal image datasets. They applied the image acquisition for the screening method with an existing database. The next step was pre-processing, which helped in improving the images and removing the unwanted noise, which consisted of three steps, viz. converting color space, filtering, and enhancement of image for the quality of the retinal image. The region-based segmentation was processed to identify the boundary of the backside images. The Gabor wavelets were applied for the feature extraction approach to extract useful information from large datasets. Further, the adaptive boost classification was applied to obtain a better prediction result for the retinal images. The system experimentally reported an accuracy of 98.4%, a specificity of 98.8%, and a sensitivity of 98.4%.

Li et al. [54] developed a new optical coherence tomography system based on deep learning to diagnose diabetic retinopathy at an earlier stage. The system was validated with OCT images collected from the Wenzhou Medical University (WMU). The dataset consists of 4168 OCT images collected from 155 patients. A total of 1112 images out of the 4168 images belong to DR grade 1 and 1856 to DR grade 0. The pre-processing was performed by resizing the OCT images to 224 × 224. The OrgNet and segmentation calculated the deep characteristics to obtain an extra feature for better classifications. In this work, the feature merging was processed through the summation method in place of concatenation. The augmentation technique was processed to enhance the neural network environment. The system is provided with the DR multi-classification, such as grades 0 and 1. An accuracy of 92%, specificity of 90%, and sensitivity of 0.95 were recorded for grade 0 DR classification.

The fundus image is the perquisition stage to calculate the accurate severity rate of DR. The manual scoring procedure is considered challenging because of the dissimilarity in morphology, number, and image size. In this regard, Sambyal et al. [55] presented an automated method based on segmentation that helps detect the boundaries and helps ophthalmologists quickly detect the DR with severity grades. They developed an improved U-Net architecture inspired by U-Net [56] that is pretrained on ResNet34 [37]. It contains the encoder and decoder at their left and right parts, respectively, resulting in better system performance. This method is also useful for improving the result compared to the existing method. The system is validated on two public datasets, viz. e-ophtha [57] and IDRiD [58]. The experimental result reported 99.88% accuracy, 99.85% sensitivity, and 99.95% specificity for the IDRiD Dataset. For the e-ophtha datasets, the accuracy was 99.98%, with a sensitivity of 99.88%.

Quellec et al. [59] proposed a machine-learning-based solution for diabetic retinopathy detection at an early stage. The authors utilized heat map concepts to identify the importance of a particular pixel in an image. To produce a good quality heat map, they trained the ConvNets network with the help of the backpropagation method. Three different categories of the dataset were used in this study (i.e., Kaggle Diabetic Retinopathy, DiaretDB1 [60], and ‘e-ophtha’). The proposed method is validated on approximately 90,000 fundus images. They followed data augmentation and pre-processing processes to transform images (i.e., 448 × 448 pixels). To train the dataset, the three ConvNets were trained to detect diabetic retinopathy. The performance of the proposed model on different datasets was found to be 0.954, 0.955, and 0.949, respectively.

Liu et al. [61] proposed a weighted path CNN (WPCNN) model to detect the diabetic retinopathy with severity levels. The system was validated through the raw database comprising 60,000 images categorized into 0 and 1 on severity scales. The authors divided the datasets into 80% and 20% training and testing sets. They scaled and resized the images to 299 × 299 through the pre-processing steps. The data augmentation method was applied to fit the image at standard formation such as right, up, left side, etc. The convolution layer processed the feature extraction through CNN and extracted the noteworthy features from the retinal fundus images. During the experimental setup, the authors suggested an over-fitting issue if the size of the network expands. The coefficient of the WPCNN was enhanced by using the backpropagation method. The system experimentally reported 94.02% accuracy in comparison to the existing models. It also achieved an AUC of 0.9823 and an F1-score of 0.9087, the highest compared to the existing methods. Although this proposed system achieved a good performance, the authors pointed out that adding more features to the automated system can improve the overall performance of the system.

Hua et al. [62] introduced a trilogy of skip-connection deep networks (Tri-SDN) to analyze the DR images. The new attribute based on EMR was introduced to identify the risk probability to increase the system’s performance. In the first phase, the feature extraction was performed from the ImageNet database. The ResNet [37] is pre-trained by the multiple convolution layers. Further, the corresponding vector mapped the feature map to identify the risk factor in the DR images. The deep learning network was built with the two skip connection blocks to identify the characteristics of the retinal images. The authors also applied the EMR-based value to identify the risk factor of the severity because it provides the numerical value, and in this work, 22 risk factors are involved. The EMR-based value is used for the DR orientation characteristic to improve the performance. The system was validated with the historical information of the 96 patients collected from the medical university in South Korea. The system experimentally reported an accuracy of 90.6%, a sensitivity of 96.5%, and an of 88.8% of AUROC, which is higher than the existing models such as random forest and 11-layer CNN. Although the system provides a good performance compared to the existing algorithm, it needs to add more retinal images to make the system more efficient so that the ophthalmologists can make easy decisions.

Reddy et al. [63] claimed that DR could be easily detected through different machine-learning algorithms. For this experiment, they used the DIARETDb1 [60] data set containing 89 images, out of which 5 are of the normal stage, and the rest of the 84 images are Mild Non-proliferative DR (NPDR) cases. The pre-processing was achieved through the grey scaling method, image copper, and image resizing to remove the noise and improve detection accuracy. They applied the segmentation technique to visualize the blood vessels in the retina. Further, the region growing technique was utilized to identify whether the pixels belong to the same region or different regions. The clustering method was applied for the data analysis. Feature extraction was applied to generalize and extract different features from the data sets. For classifications, the authors employed the SVM, k-NN, and probabilistic neural network (PNN) techniques. Different matrices such as accuracy, TPR, and FPR were employed to evaluate different classifiers. They experimentally determined the best accuracy (96.57%) through cross-validation using SVM.

**Figure 4:**
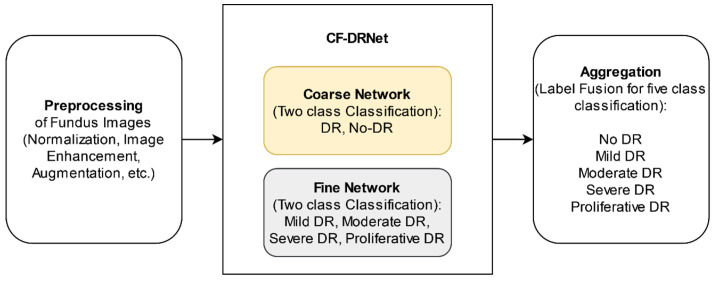
Coarse–Fine Diabetic Retinopathy Network [64].

Wu et al. [64] proposed an automated hierarchically Coarse-to-Fine network (CF-DRNet) tool to detect the DR, as depicted in Figure 4. They applied a convolution neural network to classify severity, viz. no DR, mild DR, moderate DR, severe DR, and proliferative DR. The experiment was performed on 88,400 fundus image datasets taken from Kaggle. This technique integrates three steps in which the first step performed the pre-processing, the second phase performed the CF-DRNet module, and the last stage performed the aggregation concept. The pre-processing was performed through image enhancement, image normalization, and data augmentation. Image enhancement is applied to remove unwanted noise with varying luminous factors. Image normalization is used to reduce the complexity and normalize the pixels of images in the coarse network. Data augmentation is performed to reduce over-fitting and imbalance issues in the datasets. Further, the CF-DRNet is applied to check the presence of DR. For better detection, it is classified into two different networks, such as the coarse and fine networks. Then, the aggregation method is applied to determine the level of DR and No DR. The authors experimentally claimed that CF-DRNet reported the highest accuracy of 83.10%, sensitivity of 53.99%, and specificity of 91.22%.

The detection of diabetes with different severity levels is a complicated system; hence, it is very difficult and time-consuming. In this regard, Pratt et al. [65] proposed a machine-learning-based approach with 75% accuracy for the diagnosis of diabetes in five levels of severity classifications. To train the network, the KAGGLE dataset with 80,000 retinal images was utilized. The color normalization has been processed through OpenCV for the categorization of the data into a different age, group, and authenticity. Further, they resized the images into 512 × 512 pixels for the identification of complex features. Stochastic gradient descent was utilized for training the datasets with a 0.0001 learning rate for five epochs. To concede, the first 10,290 images were pre-trained through the CNN network to classify the severity levels. Further, 5000 images took 188 s for the validation process. This technique achieved a specificity and sensitivity of 95% and 30%, respectively.

On the other hand, Yun et al. [66] proposed a backpropagation method to classify the DR into four categories, viz. normal, severe, moderate, and proliferative DR. The authors used 124 retinal images from Singapore University to process the work. This method was trained with 27 samples as training sets and the remaining as testing data samples. A feed-forward neural network was utilized to classify images into different classes. The pre-processing of images has been carried through the histogram and binarization process. Further, the ANOVA process extracted the features of retinal images into different areas and categories. The authors evaluated the model’s performance on three matrices, i.e., accuracy, specificity, and sensitivity. The method reported 80% accuracy, 90% sensitivity and 100% specificity.

Akram et al. [67] developed a multi-model for categorizing severity levels of DR into normal, mild, moderate, and severe Non-Proliferative Diabetic Retinopathy (NPDR). This model is the hybrid of medoids and the Gaussian Mixture Model (GMM) for the best classification and solving the overfitting issue. The mean-based approach was utilized to remove the noise and background. The segmentation process has been processed through Gabor and the multi-layer thresholding processes. For processing, the authors utilized datasets such as DRIVE and STARE, which are easily available in the public domain. They divided the datasets into two parts: an image and a lesion. Further, the feature vector was used to classify the severity of NPDR through color and intensity factors. The performance was evaluated on accuracy, sensitivity, specificity, and AUC metrics. The model reported 97.56% accuracy and 97.39% sensitivity, with 98.02% specificity.

Mookiah et al. [68] proposed a system for automated classification of normal, Non-Proliferate Diabetes Retinopathy (NPDR) and Proliferated Diabetes Retinopathy (PDR) using retinal images. They applied pre-processing techniques such as Wiener filtering, gray level shading correction using low pass filtering, and contrast enhancement to remove noise and uneven illumination. They also removed the optical disk to reduce the number of false positives reported while detecting the lesions. The authors applied A-IFS Histon and the 2D Gabor-matched filter approach for segmentation. Further, they extracted the features such as blood vessel area, exudate area, bifurcation point count, Local Binary Pattern (LBP) energy, LBP entropy, Laws mask energy, and entropies from the fundus images. The authors employed Probabilistic Neural Network (PNN), Decision Tree (DT), and SVM for the classification. They applied genetic algorithm and Particle Swarm Optimization (PSO) algorithms to optimize the efficacy of the classifiers. The authors experimentally determined the threshold value as 0.0104 and claimed that PNN reports the highest accuracy of 96.15%, sensitivity of 96.27%, and specificity of 96.08%.

Chowdhury et al. [69] developed a method to detect DR through four levels categorized as normal, PDR, average PDR, and acute PDR. The categorization into four levels was completed through a random forest classifier. The pre-processing was achieved through a contrast enhancement technique which helped in extracting the RGB value from 120 retinal images. The contrast augmentation was completed through adaptive thresholding to remove the unwanted noise. For conversion to a binary image, the global threshold technique was adopted. The authors utilized a feed-forward neural network based on three-layer architecture. This technique reported 90% accuracy in normal cases, 87.5% accuracy in the case of acute NPDR cases, and 90% sensitivity and 100% specificity classification. Table 2 depicts the comparison of a few works based on the severity identification for Diabetic Retinopathy. On the other hand, Kaur et al. [70] presented a systematic survey of computational methods for DR diagnosis based on fundus image analysis.

### Discussion

As mentioned earlier, early detection of the DR can help patients to recover quicker. In this regard, most of the work toward DR detection involves extracting features and classification using SVM and machine-learning-based models. The level of severity has defined various stages of the disease. Based on the literature analysis, it can be stated that although various techniques are still used to detect the disease, there is a need to improve the system in terms of complexity, detection time, and severity stages. Many existing techniques had worked on small datasets, and most of the algorithms did not elaborate on the method of feature extraction approaches. Therefore, there is a need to develop a hybrid as well as an efficient computed model to identify the severity of the DR disease at an earlier stage.

## 4. Severity Identification of Some Other Diseases

Infectious diseases, e.g., Tetanus and Hand Foot and Mouth Disease (HFMD), have a significant influence on the low- and middle-income countries [3]. Mortalities due to infectious diseases are associated with Autonomic Nervous System Dysfunction (ANSD). In addition to clinical examinations, the development of some automated computerized system is essential for the severity analysis of ANSD. In this regard, Tadesse et al. [3] presented an automated system to diagnose the severity of HFMD based on the fusion of multi-modal physiological data collected via low-cost wearable devices. For rapid diagnosis of severity levels of HFMD, their multi-layer decision system comprises an on-site triage process followed by a longitudinal model and the fusion of a multi-modal framework. Finally, deep-learning-assisted mapping of time-series physiological signals with images was obtained using spectrogram representations.

**Figure 5:**
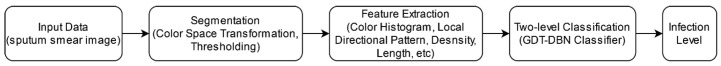
Block diagram of GDT-DBN classification for TB infection level identification [4].

Mithra and Emmanuel [4] proposed a Gaussian Decision Tree-based Deep Belief Network (GDT-DBN) for the detection of the degree of infection in the patients of Tuberculosis (TB), as depicted in Figure 5. This network is the hybrid of a Deep Belief Network (DBN), Decision Tree (DT) and Gaussian model. Initially, the sputum smear image was used as an input to the system, followed by color space transformation. For segmentation, thresholding-based mechanism was adopted. Once the segmentation is achieved, the important features (e.g., length density, local direction pattern, histogram, etc.) were extracted. The authors used the ZNSM-iDB [71] dataset comprising microscopic digital images for training and testing of the model. A two-level classification was achieved using the proposed GDT-DBN classifier. However, it is ineffective in distinguishing abnormal mycobacteria from mycobacteria TB substances due to a similarity in their geometrical structure. As mentioned earlier about the immense popularity of deep-learning-based approaches in severity identification, Alebiosu et al. [72] presented a novel DAvoU-Net segmentation framework for improving the severity assessment of tuberculosis. Experimental evaluations on the ImageCLEF 2019 TB dataset showed promising results as compared to seven other models under consideration.

Sepsis is a fatal disease if not detected at an early phase. Sequential Organ Failure Assessment (SOFA) is used to determine the level of Sepsis, but this method is totally dependent upon the laboratory measurements. In this regard, Aşuroğlu [5] presented a regression-based method to detect the level of sepsis. They used the Mart In Intensive Care (MIMIC)-III dataset [73] for experimental evaluations and binary classification for the prediction of sepsis. This model consumed less time and provided an AUC of 0.98, which is higher than other existing models. However, due to the large number of samples in the dataset, it seems difficult to balance the sepsis and un-sepsis samples, thereby causing a delay.

COVID-19 is a contagious disease that has spread all over the world, affecting the human body and health, and as such, it is very necessary to identify the level of severity at an early stage. Deep-learning-based approaches proved to be significant in the diagnosis of COVID-19 at earlier stages. CT-Scans are helpful in providing information about the severity of COVID-19 patients in medical reports. Cai et al. [74] presented a deep-learning-based approach for recognition of the infection region. Initially, patient data (RT-PCR, CT Samples) were collected and examined at different levels of severity, i.e., moderate, severe, and critical. In addition, the clinical data, including routine blood tests, clinical symptoms, demographic data, and treatments, etc., were also considered for the same reason. The 3DQI tool [75] was utilized for lesion quantifications, followed by data analysis with respect to disease severity and clinical outcomes. Chi-squared test, Student’s *t*-test and other ML models are applied for the analysis of clinical data. Two U-Net models were employed for performance analysis on 99 chest CT scans. The mean Dice Similarity Coefficient (DSC) is found to be 0.981 for lung segmentation and 0.778 for lesion segmentation. On the other hand, Yao et al. [76] proposed a machine-learning-based model to detect the severity of COVID-19. The level of severity of COVID-19 in a person is recognized by SVM with 32 features. The algorithm was used on 137 COVID-19 patients, which were confirmed by Huazhong University. Among this dataset, only 17 patients were diagnosed with mild cases, 45 cases were diagnosed with moderate cases, and the remaining 75 patients were severely infected by this disease. The samples were categorized into 80% of testing and 20% of training sets. Feature extraction has been processed through the conservative recursive features (cREF) technique to enhance the performance of the model by eliminating redundant features. The model exhibited 81% accuracy and 0.699 specificity. Roy et al. [77] presented a novel deep-learning model called Reg-STN (Regularized-Spatial Transformer Network) based on Spatial Transformer Networks (STNs) [78] for analyzing Lung Ultrasonography (LUS) images. Disease severity was predicted for each input frame of LUS images. Each frame of the LUS image was classified into four different severity levels. In addition to this, they implemented a fully annotated database called “Italian COVID-19 Lung Ultrasound DataBase (ICLUS-DB)” [79] that consists of four-level scale labels. STNs are composed of three components: (i) a localization network that is responsible for the prediction of affine transformations, (ii) a grid generator for selecting grid coordinates from images, and (iii) a sampler for wrapping the input image. The evaluation of their method was conducted for accurate prediction and localization of COVID-19 at both the frame level and video level. On the other hand, Lai et al. [80] presented a combination of ML- and DL-based approaches for detecting novel coronavirus-infected pneumonia (NCIP) from CT images. Their model is based on a few-shot learning approach. For the segmentation of lung regions from CT images, a pre-trained network is utilized. Segmentation not only reduces the lesion detection but also the computation time, thereby avoiding false positives. For lesion detection and prediction, a multitask DCNN based on U-Net was utilized. Experimental results on a real patient’s data revealed Area Under the Curve (AUC) of 0.91. Fouzia Altaf et al. [81] introduced a transfer learning concept by implementing augmented ensemble transfer learning that gives better results as compared to conventional transfer learning. To implement an efficient deep transfer learning model, they also modified the architecture of the existing network by adding an extra layer to change the dimensionality between the input image and the target image. They tested their model on the pre-trained ImageNet model. The authors used two different publicly available datasets for their execution purpose, namely Chest-Xray 14 radiographs and COVID-19 radiographs. Results on the Chest-Xray 14 dataset indicated a 50% reduction in the error rate compared to the baseline transfer learning technique. Another dataset was used for a binary problem as well as a multi-class classification problem. The modified trained model secured a 99.49% accuracy for the binary classification and 99.24% accuracy for multi-class classification. Zekuan Yu [82] identified 19 severity levels in CT scans through the classification of deep features. A total of 729 2D axial plan slices with 246 severe cases and 483 non-severe cases were employed in this study. By taking advantage of the pre-trained deep neural network, four pre-trained off-the-shelf deep models (Inception-V3, ResNet-50, ResNet-101, DenseNet-201) were exploited to extract the features from these CT scans. To identify the severe and non-severe COVID-19 cases, the features were then fed to multiple classifiers. Three validation strategies (holdout validation, tenfold cross-validation, and leave-one-out) were employed to validate the feasibility of the proposed pipelines. Experimental evaluations represented promising results as the DenseNet-201 with cubic SVM model achieved the best performance. Specifically, it achieved the highest severity classification accuracy of 95.20% and 95.34% for 10-fold cross-validation and leave-one-out, respectively. The established pipeline was able to achieve a rapid and accurate identification of the severity of COVID-19. This may assist physicians in making more efficient and reliable decisions. Many other works on COVID-19 diagnosis using AI-based approaches [83,84,85] were published by researchers. Chahar et al. [86] and Sinwar et al. [87] presented a survey of such learning models.

**Figure 6:**
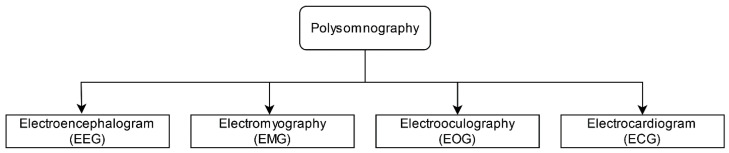
Different categories to measure the sound using polysomnography [88].

Taehoon Kim et al. [88] implemented a machine-learning model to identify the severity of sleep disorder breathing (sleep apnea). As a dataset, they considered patients that were presented at a sleep center with snoring while breathing during sleep. The authors developed four categories (i.e., normal, mild, moderate, and severe) of severity based on their Apnea Hypopnea Index (AHI) value among 120 patients. To capture the breathing sound, they used polysomnography, which records the sound using four different methods, as mentioned in Figure 6.

The recorded sound also had some noise components (i.e., machine noise, conversion noise); thus, the authors utilized two different filtering processes (i.e., spectral subtraction filtering and sleep stage filtering) to capture the useful information from the recorded sound. After filtering the sound, various audio features were extracted, and then four group and binary classification algorithms were applied to it. As a result, they scored 88.3% accuracy for the four-stage classifier and 92.5% accuracy for binary classification.

Linda A. Antonucci et al. [89] illustrated a machine-learning model to identify psychosis at an early stage. To implement the model, the authors used a support vector classifier and cross-validation section. They trained the model on approximately 105 samples composed of 71 samples of healthy controls, and 34 were psychosis samples. A total of three tests were evaluated on the samples, namely the discovery sample (healthy controls vs. psychosis), clinical validation sample (healthy controls vs. early stage of disease), and validation of familial risk (healthy controls vs. familial high risk). The resultant accuracy achieved for all three above-mentioned tests was found to be 72.2%, 63.5%, and 44.2%, respectively. The performance of the system may be improved with the help of a large dataset because a small dataset may lead to an overfitting issue.

Ahmad Abujaber et al. [90] implemented two different machine-learning models (i.e., linear regression and Artificial Neural Network) to predict the severity level of traumatic brain injury. The authors included 785 patients’ (581 survived and 204 deceased) data as a dataset in their research. Pre-processing steps were also applied to the gathered dataset in the form of cleaning and transformation. The trained model achieved an accuracy of 87% using LR and 80.9% using ANN. In the end, they concluded that the LR model provided good results compared to an ANN.

Zeng Z et al. [91] identified local recurrences in breast cancer using Electronic Health Records (EHRs). They reviewed the development corpus of 50 progress notes and extracted partial sentences that indicated breast cancer local recurrence. MetaMaps were used to process these partial sentences to obtain a set of Unified Medical Language Systems (UMLS). After using MetaMaps on patients’ progress notes, the sets that came under positive concept sets were retained. An SVM was trained to identify the local recurrences using these features with the pathology records of each patient. The model was compared with three baseline classifiers using either full MetaMap concepts, filtered MetaMap concepts, or bag of words. The model achieved the best AUC of 0.93 in cross-validation and 0.87 in held-out testing. This model provides an automated way to identify local breast cancer recurrences as compared to a labor-intensive chart review. By minimally adapting the positive concept set, the study can be replicated at other institutions with a moderately sized training dataset.

Kwon et al. [92] presented an automated classification of Knee Osteoarthritis (KOA) by combining both deep-learning and machine-learning approaches. Their automated system is based on the Kellgren–Lawrence (KL) grading system, gait analysis data, and radiographical images. Inception-ResNet-v2 was utilized for extracting relevant features from radiographical images followed by KOA multi-classification using SVM. Experimental results on both radiographical images and gait data indicated that both radiographical images and gait data are complementary for KOA classification.

Alzheimer’s disease (AD) is one of the most common neurodegenerative diseases in the world. Currently, the diagnosis of AD is carried out using Mini-Mental State Exam (MMSE), which is quite a complex and time-consuming process. Martinez-Murcia et al. [93] presented an autoencoder-based deep-learning methodology to find out the relationship between neurodegeneration and cognitive symptoms. For the analysis and visualization of distortion of extracted features, regression and SVM-based classification techniques were employed. Experimental results on the ADNI dataset revealed the classification accuracy to be 84%. On the other hand, Sethuraman et al. [94] evaluated the severity of Alzheimer’s Disease using Biomarkers. They utilized an ADNI Dataset [95] that comprises neuroimages of persons affected by AD. Their deep-learning-based model showed a performance accuracy of 96.61%.

### Discussion

Computational intelligence-based methods are used in a variety of ways to strengthen the medical field. It is hard to imagine the existence of the medical field and the subsequent treatment of several critical diseases without CI-based methods. In this section, a critical review of various CI-based methods for identifying the severity of diseases is presented. A variety of diseases (e.g., COVID-19, sleep disorder, psychosis, brain diseases, breast cancer, knee osteoarthritis, sepsis, tuberculosis, etc.) are covered for severity identification by different researchers using various techniques (deep belief networks, decision tree, Chi-squared test, Student’s *t*-test, regression, deep learning, etc.). The performance of these systems on a single type of data (e.g., imaging data, sensor data, etc.) is found to be satisfactory. However, in the future, hybrid systems (comprising several types of data as well as an ensemble of several techniques) need to be deployed to strengthen the medical field.

## 5. Some Public Repositories for Disease Severity Identification

The dataset plays a very crucial role in analyzing the performance of disease identification methods. Table 3 presents some public repositories that can be utilized to conduct disease severity identification tasks, mainly on DR, PD, and COVID-19.

The EyePACS dataset [51] is found to be one of the famous datasets for performing DR identification. It consists of approximately five million retinal images captured on different degrees of DR. In addition to retinal images, fundus images are also playing vital roles in the identification of diabetic retinopathy. MESSIDOR [58], IDRiD fundus [96], DIARETDB0 [96], DIARETDB1 [97], and E-ophtha [16] are a few famous repositories that contain fundus images to accomplish DR identification tasks. To perform COVID-19 identification from chest X-ray and CT-scan images at an early stage, several COVID-19 datasets [105,106,107,108,109,110] were made available to the public.

## 6. Conclusions

There is no doubt that on-time disease severity identification can save the lives of human beings. Many researchers have used artificial intelligence and machine-learning-based techniques to identify the severity level of different categories of diseases based on their symptoms. The study embodied in this paper was focused mainly on two diseases, viz. Parkinson’s Disease and Diabetic Retinopathy. However, severity identification of a few other diseases, such as COVID-19, autonomic nervous system dysfunction, tuberculosis, sepsis, sleep apnea, psychosis, traumatic brain injury, breast cancer, knee osteoarthritis, and Alzheimer’s disease, was also briefly covered. For severity identification, the task of multi-level classification was adopted. Based on patterns in the input data, the multiple output classes indicated different severity levels of the disease. Hoehn (H) and Yahr (Y), through the UPDRS measure, was found to be utilized mainly for severity identification. It was observed from the literature on Parkinson’s Disease (PD) that there is a huge scope to improve the accuracy using non-motor symptoms. On the other hand, for severity identification of Diabetic Retinopathy (DR), a scope to reduce the algorithmic complexity and detection rate was observed. For rapid diagnosis of COVID-19, researchers applied various models (e.g., Inception-V3, ResNet, DenseNet, etc.) to a patient’s X-ray and CT scan images. This article also provided the information of some public repositories for conducting disease severity identification tasks on DR, PD, and COVID-19. It is evident that that deep-learning models provide several advantages, viz. rapid diagnosis of diseases, automatic feature extraction, learning from examples, etc. In addition to these, they also suffer from several drawbacks, viz. lack of transparency, inefficiency in processing low-quality images, a massive amount of data required for better accuracy, etc. It can be stated that not only the development of automated disease severity identification is in its infancy stage, but also the development of massive as well as hybrid datasets enriched with epidemic characteristics. There is no doubt that deep-learning approaches have the capability of rapid diagnosis of disease, but imaging data alone do not serve this purpose. Thus, the integration of clinical and statistical observations with computational intelligence-based approaches is essential not only for an enhancement in the accuracy of computations, severity identification, and subsequent validation of results but also for minimizing outbreaks.

## Figures and Tables

**Table 1:** Performance analysis of various Parkinson’s Disease (PD) identification approaches.

References	Input	Features Extraction Approach	Classifier	Performance Accuracy (%)
Pereira et al. (2016) [10]	Spiral, Meander images	Zhang–Suen-based thinning algorithm	NB, OPF, SVM	67.00
Cantürk (2021) [26]	Gait Signals	Alexnet	SVM, kNN	99.00
Xia et al. (2019) [8]	Gait information	CNN 2D	CNN & LSTM	99.31
Zhao et al. (2018) [27]	Gait information	CNN model	CNN & LSTM	97.86
Hariharan et al. (2014) [22]	Speech samples	PCA, LDA, SFS	LS-SVM, PNN, and GRNN	100.00
Prashanth et al. (2014) [11]	SPECT images	LR	SVM, LR	96.14
Sztaho et al. (2017) [20]	Speech Rhythm	Feature Vector	SVM, Deep learning	94.87
Maachi et al. (2020) [29]	Gait signals	Manual method	Deep 1D-convent	98.70
Lahmiri and Shmuel (2019) [13]	Voice pattern	Wilcoxon-based	SVM	92.21
Ertuǧrul et al. (2016) [14]	Gait signals	1D-LBP	LR, MLP, NB, BAyesNT	88.90
Yurdakul et al. (2020) [34]	Gait Signals	Local Binary Patterns	Generalized Linear Regression Analysis (GLRA) and SVM	98.30
Oung et al. (2018) [25]	Speech and Motion signal	Wavelet Energy and Entropy	kNN, PNN, ELM	95.93
Prashanth and Roy (2018) [30]	Motor signals	Wilcoxon rank-sum test	SVM, Random Forest, probabilistic ADAboost-based ensemble	97.46
Aydın and Aslan (2021) [32]	Gait Pattern	One R Attribute Evaluation and vibes algorithm	Hilbert-Huang transform	98.79
Kim et al. (2018) [24]	Wrist sensor pattern	Convolutional filters of CNN	CNN	85.00
Balaji E. et al. (2020) [23]	Gait signals	Statistical analysis	DT, BC, EC and SVM	99.50

**Table 2:** Performance analysis of some diabetic retinopathy identification approaches.

References	Input Image	Features Extraction Approach	Classifier	Performance Accuracy (%)
Hua et al. (2019) [62]	Fundus Images	ResNet 50	Tri-SDN	90.60
Wu et al. (2020) [64]	Fundus Images	Resnet	CF-DRNet	83.10
Pratt et al. (2016) [65]	Fundus Images	PCA	CNN	75.00
Chowdhury et al. (2019) [69]	Fundus Images	Feature Vector	RF, NB	93.58
Li et al. (2019) [54]	OCT images	Org_Net and Seg_Net	OCTD_Net	92.00
Hacisoftaoglu et al. (2020) [48]	Fundus images	Not mentioned	SVM, NB, RF	98.60
Akram et al. (2014) [67]	Fundus Images	Gabor filter	GMM and m-Mediods	97.56
Mookiah et al. (2013) [68]	Fundus images	LBP, LTE	PNN, DT, SVM	96.15
Sambyal et al. (2020) [55]	DR Images	ResNet 34	Modified U-net with ResNet	99.88
Liu et al. (2019) [61]	Fundus images	WP-CNN	WP-CNN	94.23
Washburn et al. (2020) [53]	Color retinal images	Gabor wavelets	AdaBoost	98.40
Yun et al. (2008) [66]	Retinal optical images	Imaging technique	Neural Network	84.00

**Table 3:** Public datasets available for conducting disease severity identification tasks.

Contributor	Name of Database	Modality	Disease
EyePACS [45]	EyePACS	Fundus Images	DR
Decencière et al. [51]	MESSIDOR	Fundus Images	DR
Porwal et al. [58]	IDRiD fundus	Fundus Images	DR
Kauppi et al. [96]	DIARETDB0	Fundus Images	DR
Kauppi 2007 [96]	DIARETDB1	Fundus Images	DR
S. R. Rath [97]	Diabetic Retinopathy	Fundus Images	DR
Chalakkal et al. [98]	UoA-DR	Fundus Images	DR
J. Staal et al. [99]	DRIVE	Fundus Images	DR
M. Goldbaum [100]	STARE	Fundus Images	DR
Decencière [101]	E-ophtha	Fundus Images	DR
Clayton et al. [102]	HandPD	Handwriting images	PD
Goldberger et al. [9]	PhysioNET	Spiral & meander image	PD
Alam et al. [103]	VGRF	Gait information	PD
Acharya et al. [16], University of Bonn [104]	EEG time series data	EEG Signals	Epilepsy
ICLUS [79]	ICLUS—Italian COVID-19 Lung Ultrasound project	Ultrasound	
Cohen et al. [105]	COVID-19 image data collection	X-ray, CT	COVID-19 and other associated diseases
Xuehai He et al. [106]	CT-Dataset: a CT scan dataset about COVID-19	CT	COVID-19
Wang et al. [107]	COVIDx	X-ray	COVID-19, Pneumonia, Normal
RSNA [108]	COVID-19 Imaging Data Sets	X-ray, CT	COVID-19, Pneumonia
Chowdhury et al. [109,110]	COVID-19 Radiography Database	X-ray	COVID-19, Pneumonia, Normal
Eduardo Soares et al. [111]	SARS-CoV-2 CT-scan dataset	CT	COVID-19, Normal

## Data Availability

All data are included in the main manuscript.

## References

[1] Wu D., Gong K., Arru C.D., Homayounieh F., Bizzo B., Buch V., Ren H., Kim K., Neumark N., Xu P. (2020). Severity and consolidation quantification of COVID-19 from CT images using deep learning based on hybrid weak labels. IEEE J. Biomed. Health Inform..

[2] Nguyen H.H., Saarakkala S., Blaschko M.B., Tiulpin A. (2020). Semixup: In-and out-of-manifold regularization for deep semi-supervised knee osteoarthritis severity grading from plain radiographs. IEEE Trans. Med. Imaging.

[3] Tadesse G.A., Javed H., Thanh N.L.N., Thi H.D.H., Thwaites L., Clifton D.A., Zhu T. (2020). Multi-modal diagnosis of infectious diseases in the developing world. IEEE J. Biomed. Health Inform..

[4] Mithra K., Emmanuel W.S. (2021). Gaussian model based hybrid technique for infection level identification in TB diagnosis. J. King Saud Univ.-Comput. Inf. Sci..

[5] Aşuroğlu T., Oğul H. (2021). A deep learning approach for sepsis monitoring via severity score estimation. Comput. Methods Programs Biomed..

[6] Zhang Z., Sejdić E. (2019). Radiological images and machine learning: Trends, perspectives, and prospects. Comput. Biol. Med..

[7] MedlinePlus Imaging and Radiology. https://medlineplus.gov/ency/article/007451.htm.

[8] Xia Y., Yao Z., Ye Q., Cheng N. (2019). A dual-modal attention-enhanced deep learning network for quantification of Parkinson’s disease characteristics. IEEE Trans. Neural Syst. Rehabil. Eng..

[9] Goldberger A.L., Amaral L.A., Glass L., Hausdorff J.M., Ivanov P.C., Mark R.G., Mietus J.E., Moody G.B., Peng C.K., Stanley H.E. (2000). PhysioBank, PhysioToolkit, and PhysioNet: Components of a new research resource for complex physiologic signals. Circulation.

[10] Pereira C.R., Pereira D.R., Silva F.A., Masieiro J.P., Weber S.A., Hook C., Papa J.P. (2016). A new computer vision-based approach to aid the diagnosis of Parkinson’s disease. Comput. Methods Programs Biomed..

[11] Prashanth R., Roy S.D., Mandal P.K., Ghosh S. (2014). Automatic classification and prediction models for early Parkinson’s disease diagnosis from SPECT imaging. Expert Syst. Appl..

[12] Cernak M., Orozco-Arroyave J.R., Rudzicz F., Christensen H., Vásquez-Correa J.C., Nöth E. (2017). Characterisation of voice quality of Parkinson’s disease using differential phonological posterior features. Comput. Speech Lang..

[13] Lahmiri S., Shmuel A. (2019). Detection of Parkinson’s disease based on voice patterns ranking and optimized support vector machine. Biomed. Signal Process. Control.

[14] Ertuğrul Ö.F., Kaya Y., Tekin R., Almalı M.N. (2016). Detection of Parkinson’s disease by shifted one dimensional local binary patterns from gait. Expert Syst. Appl..

[15] Marek K., Jennings D., Lasch S., Siderowf A., Tanner C., Simuni T., Coffey C., Kieburtz K., Flagg E., Chowdhury S. (2011). The Parkinson progression marker initiative (PPMI). Prog. Neurobiol..

[16] Acharya U.R., Molinari F., Sree S.V., Chattopadhyay S., Ng K.H., Suri J.S. (2012). Automated diagnosis of epileptic EEG using entropies. Biomed. Signal Process. Control.

[17] Nilashi M., Ahmadi H., Sheikhtaheri A., Naemi R., Alotaibi R., Alarood A.A., Munshi A., Rashid T.A., Zhao J. (2020). Remote tracking of Parkinson’s disease progression using ensembles of deep belief network and self-organizing map. Expert Syst. Appl..

[18] Awad M., Khanna R. (2015). Support vector regression. Efficient Learning Machines.

[19] Jang J.S. (1993). ANFIS: Adaptive-network-based fuzzy inference system. IEEE Trans. Syst. Man Cybern..

[20] Sztahó D., Tulics M.G., Vicsi K., Valálik I. Automatic estimation of severity of parkinson’s disease based on speech rhythm related features. Proceedings of the 8th IEEE International Conference on Cognitive Infocommunications (CogInfoCom 2017).

[21] Park D.H., Kim H.K., Choi I.Y., Kim J.K. (2012). A literature review and classification of recommender systems research. Expert Syst. Appl..

[22] Hariharan M., Polat K., Sindhu R. (2014). A new hybrid intelligent system for accurate detection of Parkinson’s disease. Comput. Methods Programs Biomed..

[23] Balaji E., Brindha D., Balakrishnan R. (2020). Supervised machine learning based gait classification system for early detection and stage classification of Parkinson’s disease. Appl. Soft Comput..

[24] Kim H.B., Lee W.W., Kim A., Lee H.J., Park H.Y., Jeon H.S., Kim S.K., Jeon B., Park K.S. (2018). Wrist sensor-based tremor severity quantification in Parkinson’s disease using convolutional neural network. Comput. Biol. Med..

[25] Oung Q.W., Muthusamy H., Basah S.N., Lee H., Vijean V. (2018). Empirical wavelet transform based features for classification of Parkinson’s disease severity. J. Med. Syst..

[26] Cantürk İ. (2021). A computerized method to assess Parkinson’s disease severity from gait variability based on gender. Biomed. Signal Process. Control.

[27] Zhao A., Qi L., Li J., Dong J., Yu H. (2018). A hybrid spatio-temporal model for detection and severity rating of Parkinson’s disease from gait data. Neurocomputing.

[28] PhysioNet: The Research Resource for Complex Physiologic Signals. https://physionet.org/.

[29] El Maachi I., Bilodeau G.A., Bouachir W. (2020). Deep 1D-Convnet for accurate Parkinson disease detection and severity prediction from gait. Expert Syst. Appl..

[30] Prashanth R., Roy S.D. (2018). Novel and improved stage estimation in Parkinson’s disease using clinical scales and machine learning. Neurocomputing.

[31] Prashanth R., Roy S.D. (2018). Early detection of Parkinson’s disease through patient questionnaire and predictive modelling. Int. J. Med. Inform..

[32] Aydın F., Aslan Z. (2021). Recognizing Parkinson’s disease gait patterns by vibes algorithm and Hilbert-Huang transform. Eng. Sci. Technol. Int. J..

[33] Saravanan S., Ramkumar K., Adalarasu K., Sivanandam V., Kumar S.R., Stalin S., Amirtharajan R. (2022). A Systematic Review of Artificial Intelligence (AI) Based Approaches for the Diagnosis of Parkinson’s Disease. Arch. Comput. Methods Eng..

[34] Yurdakul O.C., Subathra M., George S.T. (2020). Detection of parkinson’s disease from gait using neighborhood representation local binary patterns. Biomed. Signal Process. Control.

[35] Shankar K., Zhang Y., Liu Y., Wu L., Chen C.H. (2020). Hyperparameter tuning deep learning for diabetic retinopathy fundus image classification. IEEE Access.

[36] Welikala R., Fraz M., Williamson T., Barman S. (2015). The automated detection of proliferative diabetic retinopathy using dual ensemble classification. Int. J. Diagn. Imaging.

[37] He K., Zhang X., Ren S., Sun J. Deep residual learning for image recognition. Proceedings of the IEEE Conference on Computer Vision and Pattern Recognition.

[38] Szegedy C., Liu W., Jia Y., Sermanet P., Reed S., Anguelov D., Erhan D., Vanhoucke V., Rabinovich A. Going deeper with convolutions. Proceedings of the IEEE Conference on Computer Vision and Pattern Recognition.

[39] Simonyan K., Zisserman A. (2014). Very deep convolutional networks for large-scale image recognition. arXiv.

[40] Krizhevsky A., Sutskever I., Hinton G.E. (2012). Imagenet classification with deep convolutional neural networks. Adv. Neural Inf. Process. Syst..

[41] Wang J., Bai Y., Xia B. (2020). Simultaneous diagnosis of severity and features of diabetic retinopathy in fundus photography using deep learning. IEEE J. Biomed. Health Inform..

[42] Wang J., Bai Y., Xia B. (2019). Feasibility of diagnosing both severity and features of diabetic retinopathy in fundus photography. IEEE Access.

[43] Hu J., Shen L., Sun G. Squeeze-and-excitation networks. Proceedings of the IEEE Conference on Computer Vision and Pattern Recognition.

[44] De La Torre J., Valls A., Puig D. (2020). A deep learning interpretable classifier for diabetic retinopathy disease grading. Neurocomputing.

[45] Bhaskaranand M., Cuadros J., Ramachandra C., Bhat S., Nittala M.G., Sadda S., Solanki K. EyeArt+ EyePACS: Automated retinal image analysis for diabetic retinopathy screening in a telemedicine system. Proceedings of the Ophthalmic Medical Image Analysis International Workshop, OmIA.

[46] Shankar K., Sait A.R.W., Gupta D., Lakshmanaprabu S., Khanna A., Pandey H.M. (2020). Automated detection and classification of fundus diabetic retinopathy images using synergic deep learning model. Pattern Recognit. Lett..

[47] Messidor ADCIS. https://www.adcis.net/en/third-party/messidor/.

[48] Hacisoftaoglu R.E., Karakaya M., Sallam A.B. (2020). Deep learning frameworks for diabetic retinopathy detection with smartphone-based retinal imaging systems. Pattern Recognit. Lett..

[49] Son J., Shin J.Y., Chun E.J., Jung K.H., Park K.H., Park S.J. (2020). Predicting high coronary artery calcium score from retinal fundus images with deep learning algorithms. Transl. Vis. Sci. Technol..

[50] Santa Cruz J.F.H. (2021). An ensemble approach for multi-stage transfer learning models for COVID-19 detection from chest CT scans. Intell.-Based Med..

[51] Decencière E., Zhang X., Cazuguel G., Lay B., Cochener B., Trone C., Gain P., Ordonez R., Massin P., Erginay A. (2014). Feedback on a publicly distributed image database: The Messidor database. Image Anal. Stereol..

[52] Harikrishnan V., Vijarania M., Gambhir A. (2020). Diabetic retinopathy identification using autoML. Computational Intelligence and Its Applications in Healthcare.

[53] Washburn P.S. (2020). Investigation of severity level of diabetic retinopathy using adaboost classifier algorithm. Mater. Today Proc..

[54] Li X., Shen L., Shen M., Tan F., Qiu C.S. (2019). Deep learning based early stage diabetic retinopathy detection using optical coherence tomography. Neurocomputing.

[55] Sambyal N., Saini P., Syal R., Gupta V. (2020). Modified U-Net architecture for semantic segmentation of diabetic retinopathy images. Biocybern. Biomed. Eng..

[56] Ronneberger O., Fischer P., Brox T. (2015). U-net: Convolutional networks for biomedical image segmentation. Proceedings of the International Conference on Medical image Computing and Computer-Assisted Intervention.

[57] Nandy Pal M., Sarkar A., Gupta A., Banerjee M. (2022). Deep CNN based microaneurysm-haemorrhage classification in retinal images considering local neighbourhoods. Comput. Methods Biomech. Biomed. Eng. Imaging Vis..

[58] Porwal P., Pachade S., Kamble R., Kokare M., Deshmukh G., Sahasrabuddhe V., Meriaudeau F. (2018). Indian diabetic retinopathy image dataset (IDRiD): A database for diabetic retinopathy screening research. Data.

[59] Quellec G., Charrière K., Boudi Y., Cochener B., Lamard M. (2017). Deep image mining for diabetic retinopathy screening. Med. Image Anal..

[60] Kauppi T., Kalesnykiene V., Kamarainen J., Lensu L., Sorri I., Raninen A., Voutilainen R., Pietilä J., Kälviäinen H., Uusitalo H. (2007). DIARETDB1 Standard Diabetic Retinopathy Database. IMAGERET-Optimal Detect. Decis. Diagnosis Diabet. Retin..

[61] Liu Y.P., Li Z., Xu C., Li J., Liang R. (2019). Referable diabetic retinopathy identification from eye fundus images with weighted path for convolutional neural network. Artif. Intell. Med..

[62] Hua C.H., Huynh-The T., Kim K., Yu S.Y., Le-Tien T., Park G.H., Bang J., Khan W.A., Bae S.H., Lee S. (2019). Bimodal learning via trilogy of skip-connection deep networks for diabetic retinopathy risk progression identification. Int. J. Med. Inform..

[63] Reddy S.S., Sethi N., Rajender R., Mahesh G. (2020). Extensive analysis of machine learning algorithms to early detection of diabetic retinopathy. Mater. Today Proc..

[64] Wu Z., Shi G., Chen Y., Shi F., Chen X., Coatrieux G., Yang J., Luo L., Li S. (2020). Coarse-to-fine classification for diabetic retinopathy grading using convolutional neural network. Artif. Intell. Med..

[65] Pratt H., Coenen F., Broadbent D.M. (2016). Convolutional Neural Networks For Diabetic Retinopathy. Elsevier Procedia Comput. Sci..

[66] Yun W.L., Rajendra Acharya U., Venkatesh Y., Chee C., Min L.C., Ng E. (2008). Identification of different stages of diabetic retinopathy using retinal optical images. Inf. Sci..

[67] Akram M.U., Khalid S., Tariq A., Khan S.A., Azam F. (2014). Detection and classification of retinal lesions for grading of diabetic retinopathy. Comput. Biol. Med..

[68] Mookiah M.R.K., Acharya U.R., Martis R.J., Chua C.K., Lim C.M., Ng E., Laude A. (2013). Evolutionary algorithm based classifier parameter tuning for automatic diabetic retinopathy grading: A hybrid feature extraction approach. Knowl.-Based Syst..

[69] Chowdhury A.R., Chatterjee T., Banerjee S. (2019). A random forest classifier-based approach in the detection of abnormalities in the retina. Med. Biol. Eng. Comput..

[70] Kaur J., Mittal D., Singla R. (2021). Diabetic Retinopathy Diagnosis Through Computer-Aided Fundus Image Analysis: A Review. Arch. Comput. Methods Eng..

[71] Shah M.I., Mishra S., Yadav V.K., Chauhan A., Sarkar M., Sharma S.K., Rout C. (2017). Ziehl–Neelsen sputum smear microscopy image database: A resource to facilitate automated bacilli detection for tuberculosis diagnosis. J. Med. Imaging.

[72] Olayemi Alebiosu D., Dharmaratne A., Hong Lim C. (2023). Improving tuberculosis severity assessment in computed tomography images using novel DAvoU-Net segmentation and deep learning framework. Expert Syst. Appl..

[73] MIMIC-III Registry of Open Data on AWS. https://registry.opendata.aws/mimiciii/.

[74] Cai W., Liu T., Xue X., Luo G., Wang X., Shen Y., Fang Q., Sheng J., Chen F., Liang T. (2020). CT quantification and machine-learning models for assessment of disease severity and prognosis of COVID-19 patients. Acad. Radiol..

[75] MGH, HMS 3DQI: 3D Quantitative Imaging Laboratory. https://3dqi-lab.github.io/3dqi_website/.

[76] Yao H., Zhang N., Zhang R., Duan M., Xie T., Pan J., Peng E., Huang J., Zhang Y., Xu X. (2020). Severity detection for the coronavirus disease 2019 (COVID-19) patients using a machine learning model based on the blood and urine tests. Front. Cell Dev. Biol..

[77] Roy S., Menapace W., Oei S., Luijten B., Fini E., Saltori C., Huijben I., Chennakeshava N., Mento F., Sentelli A. (2020). Deep learning for classification and localization of COVID-19 markers in point-of-care lung ultrasound. IEEE Trans. Med. Imaging.

[78] Jaderberg M., Simonyan K., Zisserman A. (2015). Spatial transformer networks. Adv. Neural Inf. Process. Syst..

[79] Trento U. ICLUS—Italian Covid-19 Lung Ultrasound Project. https://www.disi.unitn.it/iclus.

[80] Lai Y., Li G., Wu D., Lian W., Li C., Tian J., Ma X., Chen H., Xu W., Wei J. (2020). 2019 Novel Coronavirus-Infected Pneumonia on CT: A Feasibility Study of Few-Shot Learning for Computerized Diagnosis of Emergency Diseases. IEEE Access.

[81] Altaf F., Islam S., Janjua N.K. (2021). A novel augmented deep transfer learning for classification of COVID-19 and other thoracic diseases from X-rays. Neural Comput. Appl..

[82] Yu Z., Li X., Sun H., Wang J., Zhao T., Chen H., Ma Y., Zhu S., Xie Z. (2020). Rapid identification of COVID-19 severity in CT scans through classification of deep features. BioMedical Eng. OnLine.

[83] Kumar A., Sinwar D., Saini M. (2021). Study of several key parameters responsible for COVID-19 outbreak using multiple regression analysis and multi-layer feed forward neural network. J. Interdiscip. Math..

[84] Devi M., Maakar S.K., Sinwar D., Jangid M., Sangwan P. (2021). Applications of flying ad-hoc network during COVID-19 pandemic. IOP Conference Series: Materials Science and Engineering.

[85] Pandey A., Kedir T., Kumar R., Sinwar D. (2022). Analyzing Effects of Temperature, Humidity, and Urban Population in the Initial Outbreak of COVID19 Pandemic in India. Data Engineering for Smart Systems.

[86] Chahar S., Roy P.K. (2022). COVID-19: A Comprehensive Review of Learning Models. Arch. Comput. Methods Eng..

[87] Sinwar D., Dhaka V.S., Tesfaye B.A., Raghuwanshi G., Kumar A., Maakar S.K., Agrawal S. (2022). Artificial Intelligence and Deep Learning Assisted Rapid Diagnosis of COVID-19 from Chest Radiographical Images: A Survey. Contrast Media Mol. Imaging.

[88] Kim T., Kim J.W., Lee K. (2018). Detection of sleep disordered breathing severity using acoustic biomarker and machine learning techniques. Biomed. Eng. Online.

[89] Antonucci L.A., Raio A., Pergola G., Gelao B., Papalino M., Rampino A., Andriola I., Blasi G., Bertolino A. (2021). Machine learning-based ability to classify psychosis and early stages of disease through parenting and attachment-related variables is associated with social cognition. BMC Psychol..

[90] Abujaber A., Fadlalla A., Gammoh D., Abdelrahman H., Mollazehi M., El-Menyar A. (2020). Prediction of in-hospital mortality in patients on mechanical ventilation post traumatic brain injury: Machine learning approach. BMC Med. Inform. Decis. Mak..

[91] Zeng Z., Espino S., Roy A., Li X., Khan S.A., Clare S.E., Jiang X., Neapolitan R., Luo Y. (2018). Using natural language processing and machine learning to identify breast cancer local recurrence. BMC Bioinform..

[92] Kwon S.B., Han H.S., Lee M.C., Kim H.C., Ku Y. (2020). Machine learning-based automatic classification of knee osteoarthritis severity using gait data and radiographic images. IEEE Access.

[93] Martinez-Murcia F.J., Ortiz A., Gorriz J.M., Ramirez J., Castillo-Barnes D. (2019). Studying the manifold structure of Alzheimer’s disease: A deep learning approach using convolutional autoencoders. IEEE J. Biomed. Health Inform..

[94] Sethuraman S.K., Malaiyappan N., Ramalingam R., Basheer S., Rashid M., Ahmad N. (2023). Predicting Alzheimer’s Disease Using Deep Neuro-Functional Networks with Resting-State fMRI. Electronics.

[95] ADNI Alzheimer’s Disease Neuroimaging Initiative. https://adni.loni.usc.edu/.

[96] Kauppi T., Kalesnykiene V., Kamarainen J.K., Lensu L., Sorri I., Uusitalo H., Kälviäinen H., Pietilä J. (2006). DIARETDB0: Evaluation database and methodology for diabetic retinopathy algorithms. Mach. Vis. Pattern Recognit. Res. Group Lappeenranta Univ. Technol. Finl..

[97] Rath S.R. Diabetic Retinopathy 224 × 224 (2019 Data). https://www.kaggle.com/sovitrath/diabetic-retinopathy-224x224-2019-data.

[98] Chalakkal R.J., Abdulla W.H., Sinumol S. Comparative analysis of university of Auckland diabetic retinopathy database. Proceedings of the 9th International Conference on Signal Processing Systems.

[99] Staal J., Abràmoff M.D., Niemeijer M., Viergever M.A., Van Ginneken B. (2004). Ridge-based vessel segmentation in color images of the retina. IEEE Trans. Med. Imaging.

[100] Goldbaum M. STructured Analysis of the Retina. https://cecas.clemson.edu/~ahoover/stare/.

[101] Decenciere E., Cazuguel G., Zhang X., Thibault G., Klein J.C., Meyer F., Marcotegui B., Quellec G., Lamard M., Danno R. (2013). TeleOphta: Machine learning and image processing methods for teleophthalmology. Irbm.

[102] Pereira C.R., Weber S.A., Hook C., Rosa G.H., Papa J.P. Deep learning-aided Parkinson’s disease diagnosis from handwritten dynamics. Proceedings of the 2016 29th SIBGRAPI Conference on Graphics, Patterns and Images (SIBGRAPI).

[103] Alam M.N., Garg A., Munia T.T.K., Fazel-Rezai R., Tavakolian K. (2017). Vertical ground reaction force marker for Parkinson’s disease. PLoS ONE.

[104] EEG Time Series Data, University of Bonn. http://www.meb.uni-bonn.de/epileptologie/science/physik/eegdata.html.

[105] Cohen J.P., Morrison P., Dao L. (2020). COVID-19 image data collection. arXiv.

[106] Zhao J., Zhang Y., He X., Xie P. (2020). Covid-ct-dataset: A ct scan dataset about covid-19. arXiv.

[107] Wang L., Lin Z.Q., Wong A. (2020). Covid-net: A tailored deep convolutional neural network design for detection of COVID-19 cases from chest X-ray images. Sci. Rep..

[108] RSNA Radiological Society of North America COVID-19 Imaging Data Sets. https://www.rsna.org/covid-19.

[109] Chowdhury M.E., Rahman T., Khandakar A., Mazhar R., Kadir M.A., Mahbub Z.B., Islam K.R., Khan M.S., Iqbal A., Al Emadi N. (2020). Can AI help in screening viral and COVID-19 pneumonia?. IEEE Access.

[110] Kaggle COVID-19 Radiography Database. https://www.kaggle.com/tawsifurrahman/covid19-radiography-database.

[111] Angelov P., Almeida Soares E. (2020). SARS-CoV-2 CT-scan dataset: A large dataset of real patients CT scans for SARS-CoV-2 identification. MedRxiv.

